# Current Insights into the Molecular Mode of Action of Seaweed-Based Biostimulants and the Sustainability of Seaweeds as Raw Material Resources

**DOI:** 10.3390/ijms23147654

**Published:** 2022-07-11

**Authors:** Neerakkal Sujeeth, Veselin Petrov, Kieran J. Guinan, Fiaz Rasul, John T. O’Sullivan, Tsanko S. Gechev

**Affiliations:** 1 BioAtlantis Ltd., Clash Industrial Estate, Tralee, V92 RWV5 County Kerry, Ireland; plant.research@bioatlantis.com (N.S.); research@bioatlantis.com (K.J.G.); crop.strengthen@bioatlantis.com (F.R.); jtos@bioatlantis.com (J.T.O.); 2 Center of Plant Systems Biology, 139 Ruski Blvd., 4000 Plovdiv, Bulgaria; vpetrov@plantgene.eu; 3 Department of Plant Physiology, Biochemistry and Genetics, Agricultural University of Plovdiv, 12 Mendeleev Str., 4000 Plovdiv, Bulgaria; 4 Department of Plant Physiology and Molecular Biology, University of Plovdiv, 24 Tsar Assen Str., 4000 Plovdiv, Bulgaria

**Keywords:** abiotic stress, biostimulants, oxidative stress, plant priming, seaweed extracts, sustainability

## Abstract

Natural biostimulants, such as seaweed extracts, can stimulate plant growth and development in both model and crop plants. Due to the increasing demands for their use in agriculture, it is crucial to ensure the sustainability of the sources from which they are produced. Furthermore, some seaweed extracts were recently shown to prime and protect from adverse environmental factors such as drought, salinity and extreme temperatures, as well as from oxidative stress. The molecular mode of action of these biostimulants has still not been fully elucidated, but there has been significant progress in this direction in the last years. Firstly, this review examines the sustainability aspects of harvesting seaweed resources as raw materials for manufacturing biostimulants and provides an overview of the regulatory landscape pertaining to seaweed-based biostimulants. The review then summarises the recent advances in determining the genetic and molecular mechanisms activated by seaweed-based biostimulants, their influence on transcriptome reconfiguration, metabolite adjustment, and ultimately stress protection, improved nutrient uptake, and plant growth and performance. This knowledge is important for deciphering the intricate stress signalling network modulated by seaweed-based biostimulants and can aid in designing molecular priming technologies for crop improvement.

## 1. Introduction

Healthy and sustainable food production, as well as environmental protection, have become top priorities in modern agriculture. Important stress factors such as heat, drought, salinity, low soil organic matter and pathogenic infestation negatively impact agricultural productivity worldwide [1,2,3]. Abiotic and biotic stresses cause significant reductions in growth, yield and marketable grade of produce, thereby resulting in considerable economic losses annually. A staggering 50–80% of global crop yield losses are currently attributed to adverse weather events and the linked abiotic stress factors ([4] and references therein). In turn, these frequently occurring adverse weather events cause increased crop susceptibility to disease and pests [5]. The FAO estimates a direct annual production loss of 20–40% in major crops worldwide due to pests and associated biotic stress-induced damage [1]. In addition to the climate change-induced challenges outlined above, stakeholders in the agri-food system value-chain are also committed to avoiding excessive and inefficient use of chemicals and fertilisers in agricultural production, to minimise environmental and health-related impacts [6].

In recent years, several eco-friendly tools and approaches have been proposed and implemented to improve production in terms of agricultural sustainability [7,8,9]. One approach has been to provide naturally derived compositions and safe inputs, known as biostimulants, to treat crop plants. Biostimulants are naturally occurring organic molecules, plant extracts, or microorganisms that can stimulate plant growth and development. Some of them even enhance yield, without compromising on quality [10,11,12].

“Plant biostimulants” are defined by the European Commission [13] as inputs to stimulate a crop’s natural nutrition processes, aimed solely at improving the crop’s nutrient use efficiency, tolerance to abiotic stress, quality traits or increasing the availability of confined nutrients in the soil or rhizosphere. These inputs stimulate plant nutrition processes independently of the product’s own nutrient content [14]. The most common biostimulants sold in the agriculture and horticulture sector include seaweed extracts, humic acids, fulvic acids, protein hydrolysates, amino acids and microorganisms [15]. The value of the global biostimulant market in 2016 was estimated to be circa (ca.) EUR 1.45 billion with seaweed extracts and humic acids holding a major share of 33.3% and 31.1%, respectively, followed by vitamins and amino acids (10.2%), microbials (9.7%), trace minerals (9.6%) and others (6%) [16]. With increased awareness regarding the adverse effects of chemically synthesised pesticides and fertilisers and growing demand for safe and sustainable products, the global biostimulant market is projected to increase to ca. EUR 2.66 billion in 2022 [16]. It can be difficult to understand the precise mechanisms activated by biostimulants due to their complex nature. Most biostimulants available on the market consist of multiple bioactive molecules and a great diversity in plant responses is observed following their application to several crop species [17]. Dose rates (low to high), mode (foliar or soil) and period of treatment, duration, plant age and developmental stage, etc., will influence the efficacy and final benefits achieved from individual biostimulant extracts. This represents both a challenge and a great opportunity for researchers to further examine biostimulants in order to understand the fundamental and innovative principles underlying these technologies.

Seaweed extracts are regarded as one of the fastest growing biostimulant products on the market [18]. They consist of multiple bioactive molecules which induce a diverse range of effects in plants such as stress reduction, improved shoot and root growth, enhanced chlorophyll synthesis, improved fruit set and yield, fruit uniformity, delay of senescence and enhanced fruit quality at harvest (reviewed in [19] and references therein). Despite the surge in publications on seaweed-based plant biostimulants in the last few years, their molecular mode of action is still not very clear. In addition, the regulatory frameworks and sustainability aspects underpinning the sourcing of the raw material required to manufacture these products have not been fully described in the literature. This review provides an overview of seaweed-based resources worldwide, the harvesting and manufacturing practices required for their sustainable use and the various regulatory frameworks underpinning these activities. It also outlines the importance of sustainable seaweed harvesting practices, which are particularly important given the increasing demand for seaweed-based biostimulants globally, and in light of existing and forthcoming national and international regulations on seaweed-based biostimulants, such as the EU Fertilising Products Regulation (EU) 2019/1009 [13]. The review also examines the latest findings from recent “molecular level” studies that identify genes and pathways activated by seaweed-derived biostimulants, which may contribute to the direct and/or indirect benefits observed in plants in terms of growth, health and productivity. Additionally, particular emphasis is placed on recent molecular, genetic and functional analyses which demonstrate that the mechanisms employed by seaweed extracts are novel and distinct from previously proposed growth hormone models. Such studies provide a strong basis for future research into the mode(s) of action of seaweed-based biostimulants, using modern “Omics” technologies and targeted functional genetics approaches.

## 2. Resources, Sustainability and Regulation of Seaweed-Based Biostimulants

### 2.1. Chemodiversity

The diversity in the biological effects of seaweed extracts is largely associated with the high level of chemical variability of algal extracts themselves and the raw materials used in their manufacture. In particular, macroalgae groups and species are known to possess a high degree of chemodiversity in terms of bioactive compounds, pigmentation and nutrient content [20]. In addition, macroalgae may exhibit seasonal variability in the levels of their bioactive constituents and chemical characteristics [21,22,23,24,25,26,27,28,29,30,31,32,33,34,35,36,37,38]. Moreover, it has also been shown that the extraction methodology employed during the manufacturing of seaweed extracts and the quantity and availability of bioactives present therein, can also give rise to biostimulants which induce differential effects on plant growth and related parameters [39,40,41].

### 2.2. Seaweed as a Raw Material for Plant Biostimulants and Other Products

***(a)***  ***Global seaweed production from harvesting wild stock sources.***

**Figure 1:**
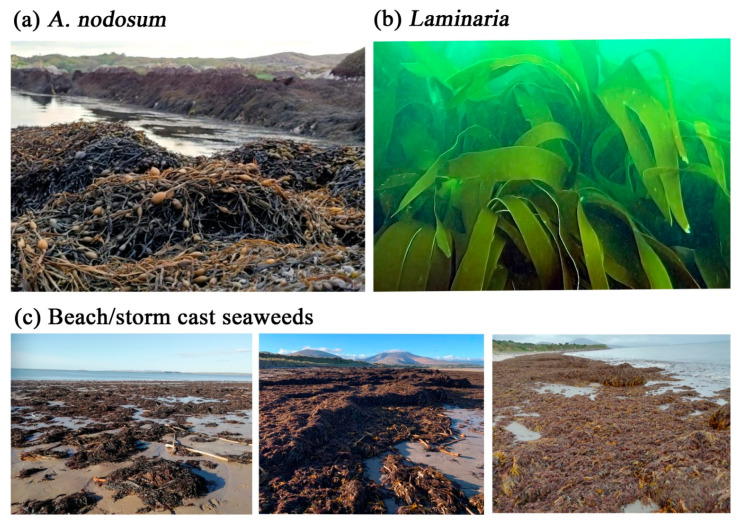
Examples of intertidal, subtidal and beach/storm-cast seaweeds. (**a**) *A. nodosum* growing in the intertidal zone in County Kerry, Ireland (source: BioAtlantis Ltd.), (**b**) *Laminaria* growing in subtidal waters of Bantry Bay, County Cork, Ireland (Crowe et al. [43]; unpublished image), and (**c**) beach/storm-cast seaweeds on Derrymore Strand, County Kerry, Ireland (source: BioAtlantis Ltd.).

Wild seaweeds are harvested as raw materials for plant biostimulant, animal feed and human food industries. Brown seaweeds are primarily used in the manufacture of biostimulants, as many grow at high density in the wild, are commercially viable and can be sustainably sourced. Figures from the Food and Agriculture Organization (FAO) indicate a total global capture production of 1,083,242 tonnes (T) of seaweeds and aquatic plants in 2019 [42]. Brown seaweeds (Phaeophyta) accounted for 62.4% (675,654 T) of the total, while the share of red seaweeds (Rhodophyta) was 17.5% (189,638 T). An undefined category (“aquatic plants, nei”) represented 18.3% of the global yield (198,617 T). Green seaweeds (Chlorophyta) contributed to 1.5% (16,230 T) of global yield, while “seaweed nei” accounted for 0.3% (3104 T; see Table 1). Examples of intertidal, subtidal and beach/storm-cast seaweeds are shown in Figure 1.

***(b)***  ***Seaweed aquaculture.***

While seaweeds are cultured worldwide ([44] and references therein), their use as raw materials for plant biostimulants may be limited. *Ascophyllum nodosum* has a low potential for production by aquaculture [45] due to its demographic characteristics [46,47], while the cultivation of bulk species such as kelp is not considered economically feasible in Europe [48], e.g., *Laminaria hyperborea*. The cultivation of seaweeds in exposed environments faces challenges due to the low survival rate of aquaculture structures and insufficiently durable equipment to withstand rough conditions ([49] and references therein). Wave heights may be problematic for offshore aquaculture in coastal areas of the North Atlantic Ocean and Norwegian Sea, (e.g., Ireland, Norway, northwest France), Chile, Namibia and South Africa [50], which experience stronger sea surface winds. Calmer conditions may be more suitable for aquaculture, for example, in regions near Indonesia, India, the North Sea and parts of the South Atlantic [50]. Life cycle and biological constraints may limit the expansion of aquaculture to commercial species. For example, *L. hyperborea* grows at lower densities in sheltered areas, which otherwise favour cultivation, and at higher density in exposed areas [51,52,53]. Thus, given the challenges faced by aquaculture in exposed environments, culturing *L. hyperborea* may not be technically feasible or commercially viable.

***(c)***  ***Beach/storm-cast seaweeds.***

There is growing interest in the use of beach/storm-cast seaweeds given their high availability and potential for developing added-value products [54]. However, the utilisation of beach-cast seaweeds in biostimulants manufacture may not always be feasible, given the regulatory and market needs for high quality end products that are free of microbial contamination. For example, decaying seaweeds can influence the survival of faecal indicator organisms in beach areas and can provide a protected environment for bacteria such as *E. coli* [55]. The association of seaweed flies in the presence of decaying seaweed beds may also facilitate environmental survival and transmission of *E. coli* [56]. Therefore, it is important that seaweed-based biostimulants are manufactured using freshly harvested and/or non-contaminated seaweed raw material.

### 2.3. Regulatory and Sustainability Aspects of Harvesting Wild Seaweed Resources

***(a)***  ***Regulatory aspects of harvesting seaweed in Europe.***

Harvesting of wild seaweeds should be undertaken in a sustainable manner, allowing for regeneration and preventing impacts on marine and coastal species and habitats [48,57,58]. In the EU, harvesting must be carried out in line with the Habitats Directive 92/43/EEC and Birds Directive 2009/147/EC, particularly when working in Natura 2000 sites, Special Areas of Conservation (SACs), Special Protection Areas (SPAs) and Marine Protected Areas (MPAs). Measures are required to ensure that conservation objectives and targets for qualifying interests (habitats, species, etc.), are not impacted [59]. Conservation objectives and targets are assigned to protect species and habitats listed in Annex I/II of the EU Habitats Directive [60,61].

In France, commercial harvesting is regulated on a national and regional scale [62]. The Parc Naturel Marin d’Iroise (PNMI), is an MPA where human activities operate according to a set of defined criteria and permits the harvesting of seaweeds such as *A. nodosum*, *L. hyperborea* and *L. digitata.* Both *L. digitata* and *L. hyperborea* are mechanically harvested on a rotational basis by boat using a gear called a “scoubidou” or a rake-like dredge, respectively. In both cases, the process appears to be sustainable as evidenced by the recovery of *Laminaria* spp. biomass post-harvesting to levels comparable with unharvested zones [63,64]. The French Research Institute for the Exploitation of the Sea monitors kelp harvesting and advises relevant administrations [62]. Most French seaweed production comes from the PNMI and Brittany [62].

The Norwegian Ministry of Fisheries and Coastal Affairs regulates seaweed harvest by-laws and instructs the Directory of Fisheries which sets the regulations together with industry, the Institute of Marine Research, researchers and other stakeholders [65]. Licenses are obtained from the Directorate of Fisheries [66] and ecological aspects of harvesting are monitored through collaboration between industry and scientists [67]. Harvesting of *L. hyperborea* occurs along 40% of Norway’s coastline and is managed on a 3-to-4-year rotational basis, typically regenerating within 4 years post-harvesting ([67] and references therein). Kelp is mechanically harvested by boat, using a 3 m wide dredge that removes plants from the substratum [66,67]. In Iceland, a new regulation on seaweed harvesting was issued in 2018 (Regulation No. 90/2018) by the Directorate of Fisheries and the Ministry of Industries and Innovation based on the Fisheries Management Act No. 116/2006 and Act No. 57/1996 concerning Treatment of Commercial Marine Stocks. Regulations specify a 4-year harvest rotation [66,68]. Species such as *L. hyperborea*, *L. digitata* and *A. nodosum* are mechanically harvested.

In Ireland, seaweed harvesting is regulated by government departments and licensed under the Foreshore Act 1933 (as amended) and the Maritime Area Planning Act 2021. License decisions are made following consultations with expert groups and prescribed bodies including the Marine Institute, National Parks and Wildlife Services, Inland Fisheries Ireland, Sea Fisheries Protection Authority, Marine Survey Office and the Underwater Archaeology Unit. Licenses are issued in line with EU regulations and involve collaboration between industry and scientists to monitor the resource. The mechanical harvesting of *L. digitata* and *L. hyperborea* in subtidal waters is a licensed activity in Bantry Bay, County Cork, Ireland and involves the cutting of kelp a minimum of 25 cm above the holdfast, without making contact with the seabed, along with scientific monitoring of kelp regeneration rates and flora and fauna 3- and 5-years post-harvesting [69].

***(b)***  ***Growth rates of seaweeds post-harvesting.***

Factors influencing the commercial viability of seaweeds include their density, availability and regeneration rates. Brown seaweeds are generally larger than green or red species and are harvested in accessible, high-density areas, either manually, (e.g., handheld blade or rake cutter) or mechanically by boat. Regeneration rates vary between species. *M. pyrifera* reaches harvestable levels within 6 months [70], while *Lessonia berteroana* (formely *L.* *nigrescens*) reaches this size within 10 months following recruitment into cleared areas [71]. At least 18 months is required for regeneration of *D. antarctica*, with recolonizing stands more uniform and compact than unharvested stands [72]. *L. trabeculata* recovery may take over two years ([73] and references therein). *H. elongata* and *F. serratus* can recover within 12 months after harvesting [74,75].

*L. hyperborea* regenerates to become the dominant species 2–3 years after harvesting, (reviewed by [76]). New *L. hyperborea* plants are observed one-year post-cutting in Scotland [77], with biomass recovering within 2.5 years in the Isle of man [78]. In Norway, *L. hyperborea* recovers between 2 to 3 years to up to 6 years post-mechanical harvesting [79]. Approximately 2.5 years after harvesting, 1- to 3-year-old plants dominate the harvest area [80]. Four years post-harvesting, *L. hyperborea* regained its dominance, due to the high density of recovering kelp vegetation [67]. Following canopy removal, understory plants flourish with the improved light conditions, ensuring a shorter regeneration time compared to recovery dependent on recruitment from spores and gametophytes. Kelp density was higher than pre-harvesting and higher than unharvested sites [67]. Five years after mechanical harvesting in France, *L. hyperborea* biomass was similar to non-harvested sites, with stipe diameter higher in the harvested area [64].

*L. digitata* coverage was regained 6 months following experimental removal in the Isle of Man [81]. In France, *L. digitata* regenerates 12–30 months post-harvesting of sporophytes [82]. Davoult, et al. [63] found no correlation between density or biomass of *L. digitata* and annual harvest quantities, concluding that harvesting can be maintained or potentially increased.

*A. nodosum* is one of the most widely used materials to manufacture biostimulants worldwide. Baardseth [83,84] determined that sustainable harvest was possible once sufficient material is left behind (reviewed by Guiry and Morrison [85]). Kelly et al. [57] reported that sites in Ireland at Clew Bay and Connemara demonstrate almost complete recovery of *A. nodosum* 11- and 17-months post-hand harvesting, respectively. In Canada, 2 to 5 years was suggested for *A. nodosum* biomass recovery ([86] and references therein), which was observed within 3 years post-removal of 50% of the biomass ([86,87] and references therein). Rapid recovery may be due to stimulation of growth and branching of the suppressed shoots of the clumps [88]. *A. nodosum* is harvested in Canada from small boats using a hand-held cutting rake [87].

Red seaweeds are harvested by hand by cutting or plucking (reviewed by [89]). *P. palmata*, *P. linearis*, *P. lucida* and *G. corneum* recover approximately 12 months post-harvesting ([74,90,91] and references therein). *G. skottsbergii* has a biennial cycle of biomass production and should be harvested in rotation [92]. *G. furcellatus* also has a relatively short regeneration time [93].

***(c)***  ***Carbon sequestration***

Although primary producers, macrophytes, (e.g., seaweeds, seagrass) account for <1% of global photosynthesis (global net primary production, NPP) [94] and about 3% of marine NPP ([95] and references therein). Non-macrophytes, (e.g., phytoplankton) contribute to >97% of marine NPP and >45% of global NPP. Land plants/habitats are responsible for >53% of global NPP [94]. As macrophytes’ share of global NPP is low, their role in carbon sequestration may be limited. Seaweeds with buoyancy mechanisms may float, degrade and sink to deep-sea sediments, for long-term carbon storage ([96] and references therein). However, European kelp species may have a lesser contribution to carbon sequestration, as large amounts of their biomass is washed ashore annually to decay as part of their life cycle. This happens to an estimated 20% of *L. hyperborea* stocks in Ireland annually, a country with approximately 3 million tonnes of standing kelp stock [97]. Given the absence of floating devices in European kelps and the nature of coastal areas of north-western Europe as receivers of decaying biomass, the long-term deep-water or sediment sequestration of their carbon may be limited. Carbon sequestration may be limited to refractory carbon associated with undisturbed beds of Fucales [96]. Approximately 12% of NPP by macroalgae may be sequestered [98], which is low given that macrophytes account for <1% of global NPP [94]. A new study also suggests that seaweed ecosystems may not mitigate CO_2_ emissions [99]. Further research is warranted, as the contribution of macroalgae to NPP may be higher in coastal areas ([95] and references therein).

***(d)***  ***Hydrodynamics***

While some field studies indicate that certain kelp may influence currents and hydrodynamics [100,101,102,103,104,105], others have not identified effects on waves or adjacent-beach width [106,107]. Morris et al. [108] reported lower wave attenuation of kelp beds compared to controls, suggesting an influence of reef substratum. Kelp species can be vulnerable to storms, which cause mortality through damage and dislodgement of entire plants, leading to thinning or clearing of kelp areas ([109] and references therein). Over 75% of *L. hyperborea* detritus is delivered as coarse material through erosion, dislodgement and spring cast of old blades [110]. Similar figures are reported for *M. pyrifera* and *E. radiata* ([110] and references therein). Kelp may be considered as susceptible to storm damage with biomass regularly lost from kelp beds and washed ashore. Vulnerability varies according to location, exposure, substratum and species ([109] and references therein). The mean age of *L. hyperborea* ranges from 3.4 years in Bantry Bay, Ireland [43], 4.6 to 7.75 years in the UK [53,111], 5.3 to 6.2 years in France [64,112], and 6 to 14 years in Norway [67,79,113]. Variability in age might be due to events such as storms, harvesting or urchin grazing [113]. The age of *L. hyperborea* in Bantry Bay [43] may reflect the rapid regeneration of kelp following its removal by Storm Darwin in 2014, one of Ireland’s worst storms on record. As kelp can regenerate at a higher density post-harvesting [67] and produce more uniform and compact stands [72], it is possible that the hydrodynamic services of kelp could be enhanced by harvesting. For example, by harvesting older plants, understory plants flourish to produce higher density kelp beds [67].

***(e)***  ***Flora and fauna***

Seaweeds reside in subtidal and intertidal marine environments which are host to a range of flora and fauna [48,57,58,114]. To ensure sustainability, harvesting must be managed to allow for regeneration and to prevent/minimise impacts. The effect of harvesting worldwide varies. Declines in *Gracilaria* spp. occurred in Hawaii, Brazil and Chile due to overharvesting [115,116,117,118,119]. In Chile, there are strategies to ensure the sustainable harvest of *Lessonia* sp. and *Macrocystis* sp. ([120] and references therein). While some studies describe impacts of *M. pyrifera* removal [121,122], others report effects limited to a small number of species and no impacts on diversity or benthic community [123,124]. Differential effects are reported for *L. trabeculata* harvesting, with increases in fish and other fauna observed 6 months post-harvesting [125].

Harvesting in European waters is largely sustainable. Commercial harvesting of *A. nodosum* is undertaken in Ireland, France, Norway and Scotland. *A. nodosum* has been hand-harvested at low tide in Ireland for decades [85] with studies indicating no impact on overall biodiversity, mobile epifauna and fish [57]; however, scientific monitoring and mitigation measures are required to ensure sustainability. In Canada, *A. nodosum* regenerates post-harvesting using the “rake” method; however, harvested biomass can contain up to 6% of holdfast material [126], potentially indicative of mortality. The presence of holdfasts in landings depends upon the underlying substrate and the quality of the cutting rakes. If the substrate is friable, *Ascophyllum* thalli can be pulled off and holdfast by-catch can go up. Dull cutting blades may increase holdfast by-catch [127]. Rake methods may be suited in areas with large, solid substratum, while hand harvesting at low tide may be preferable in regions with substrate consisting of a heterogeneous mixture of small rocks, stones, pebbles and other friable substratum.

Mechanical harvesting of kelp in Europe has been sustainable, allowing the continued harvest of *L. hyperborea* and *L. digitata* in France and Norway for over 50 years, with minimal ecological impacts. *L. hyperborea* and *L. digitata* regenerate 2 to 6 years post-mechanical harvesting [63,64,67,79,80]. Epiphyte coverage may take longer to recover [79]. While it was suggested that *Phalacrocorax carbo* foraging behaviour was altered due to harvesting, its population development was unchanged [128], suggesting no long-term effects. No alterations in the diving activity of *Phalacrocorax aristotelis* due to kelp harvesting were observed [129]. As birds such as *P. carbo* and *P. aristotelis* do not form obligate relationships with seaweed, the effects of harvesting on such species may be limited or absent. A study conducted 3 to 4 months after mechanical harvesting of *L. hyperborea* in Norway, showed no effect on total fish or total species numbers per site [130]. Positive effects on juvenile saithe were recorded, while no effects on cod or gobies were observed in harvested tracks. Crabs were unaffected as well, likely due to their association with the solid seafloor, rather than kelp itself [130]. Epiphytes and associated fauna were reduced in harvested areas and unchanged in control sites. Juvenile *Pollachius* and *Ctenolabrus rupestris* numbers were lower in harvested areas with the latter increased in control zones. Adult pollack populations were lower [130] and likely moved to other areas as their diet includes species not only found around kelp ([130] and references therein). Alterations in fish numbers may reflect movement to non-kelp sites, e.g., regions representing the broader habitat range of species for feeding or during early life, juvenile, nursery or spawning stages, such as soft bottom areas, shallow open water, saltmarsh, seagrass, oyster reef, mussel beds, rocky shores [131] and deep waters beyond the range limit of kelp habitats. Although differential effects (positive and negative) have been reported post kelp harvesting, they may be short-term and not observed once the biomass has regenerated. Since commercial fisheries species utilise a range of marine habitats [131] and do not necessarily form obligate relationships with seaweeds, effects of harvesting may be limited or absent in the long-term.

Overall, the models for seaweed harvesting in France, Norway and Ireland represent a good framework for the sustainable management of seaweed resources, particularly given their focus on collaboration between industry, harvesters and relevant scientific bodies. By regulating and managing activities and monitoring regeneration and effects post-harvesting, it is possible to undertake commercial harvesting of wild seaweed resources in a sustainable manner into the future.

### 2.4. Regulatory Aspects Governing Classification and Registration of Plant Biostimulants

In the previous decades, it was widely considered that seaweed extracts contain plant growth hormones or exhibit growth hormone-like activity. However, recent studies indicate that seaweed extracts may not contain growth hormones or impart growth hormone activity (reviewed by [132] and references therein). In addition, there has been uncertainty on the marketplace in recent times, with companies making contrasting claims regarding the mode-of-action and constituents of seaweed extracts. The variable nature of the regulatory landscape worldwide and lack of legal definitions for biostimulants has also created considerable confusion for companies and end users. To resolve these issues, the EU has added a category for “plant biostimulants” under the new fertiliser regulation, Regulation (EU) 2019/1009 [13], thus distinguishing biostimulants from plant protection products which are regulated separately (Regulation (EC) No 1107/2009 [133]). The regulation also provides a legal definition for biostimulants in terms of their nature and permitted product claims. Similar laws may be introduced in other jurisdictions worldwide to ensure that biostimulant products are governed by regulations distinct from those governing plant growth regulators, active substances, plant protection products and pesticides.

Where companies make growth hormone-type claims in relation to biostimulant or fertiliser products (their content and/or activity), this may result in misclassification or incorrect registrations. For instance, authorities in India concluded that an imported product labelled as a “seaweed extract” and used as plant growth promotor, was incorrectly classified in the category “animal and vegetable fertilisers/organic fertilisers”. Instead, the authorities indicated that the product should have been classified as a plant growth regulator [134]. In a similar case in the US [135], authorities considered that an imported product (labelled as “Liquid Seaweed Concentrate” and an “Organic Plant Nutrient”), made from *Ecklonia maxima*, was incorrectly registered as an “organic input material” and should have been registered as a pesticide with the Environmental Protection Agency. In both examples, authorities attributed the misclassification and/or incorrect registration of a product made from *E. maxima* to the presence of plant growth regulators (PGRs) or claims regarding its use as a plant growth promotor. In summary, clearly defined regulations governing the classification and registration of plant biostimulants worldwide are necessary to avoid confusion on the marketplace, misclassification and incorrect registration of plant protection products as biostimulants and vice versa.

## 3. Stimulation of Growth and Increase in Yield Induced by Seaweed Biostimulants

### 3.1. Seaweed Biostimulants and Plant Hormones

Historically, many researchers used growth hormone bioassays to examine plant growth effects observed as a result of seaweed treatments, utilising synthetic hormone as a standard (reviewed in [132] and references therein). These assays showed that seaweed applications can improve plant shoot and root growth, effects which may be comparable to those obtained by exogenous application of synthetic hormones. They described these observations as “growth hormone-like activity” induced by seaweed extracts. Such “growth hormone-like activity” terminology can be confusing and incorrectly used by companies in the current seaweed-biostimulant market. In particular, the minute levels of plant growth hormones detected in or assumed to be present in certain seaweed extracts are often speculated to be responsible for the growth effects in treated plants [132].

It has been demonstrated that in Arabidopsis, the application of an alkaline extract of *A. nodosum* can activate the cytokinin (CK)-responsive promoter of Arabidopsis *RESPONSE REGULATOR 5* (ARR5), supporting a hypothesis that such seaweed extracts (SWEs) contain compounds that may actually elicit endogenous hormone-like and hormone-inducing activity [136]. Indeed, a study by Wally et al. [137] with phytohormone biosynthetic and insensitive mutants of Arabidopsis (the *abi 4-1* mutant insensitive to abscisic acid and cytokinin, and the quadruple mutant *ipt 1,3,5,7* deficient in CK biosynthesis) demonstrated that an extract of *A. nodosum* can modulate biosynthesis, quantity and ratios of the endogenously produced cytokinin, auxin and abscisic acid (ABA) metabolites and suggested that plant phenotypic alterations (growth effects) are potentially due to endogenous hormone-associated changes, rather than due to the effects of exogenous phytohormones present within the seaweed extracts. This study also estimated the phytohormone concentrations in 12 SWEs from different sources and suggested that the phytohormone levels present in seaweed extracts were insufficient to cause significant effects in plants when applied at the recommended rates and dilutions used in the field [137].

A study by Rayorath et al. [138] investigated the potential involvement of the plant growth hormone gibberellic acid (GA_3_) in seed germination responses induced by a seaweed extract of *A. nodosum*. During seed germination in cereals, the enzyme α-amylase plays a role in hydrolysing the endosperm starch into sugars, which provides energy for the growing roots and shoots [139,140]. Endogenous gibberellins or gibberellic acids act as a signal for activating the α-amylase genes, contributing to the seed germination process [141]. Additionally, it is known that ABA, when applied on to the seeds, can negatively affect α-amylase gene induction caused by GA_3_ and can neutralise the activity of GA_3_ in germinating seeds [142]. Rayorath and co-workers [138] demonstrated that exogenous application of an alkaline extract of *A. nodosum* (ANE), without any detectable GA3 content, was able to induce α-amylase activity and seed germination of GA-deficient mutant barley seeds (*grd2*), which are unable to endogenously produce GA. This shows that compounds other than GA present in the seaweed extract are responsible for the observed effects in the barley mutant. Moreover, the α-amylase activity induced by the seaweed extract in the mutant was not inhibited by exogenous ABA applications, further confirming that non-GA compounds present in ANE act via a GA-independent pathway for seed germination in barley [138].

Another recent study by Ghaderiardakani et al. [143] further examined the roles of endogenous ABA, ethylene and cytokinin signalling in the germination and root growth responses of Arabidopsis to seaweed extract of Ulva (commonly known as sea lettuce; green nori). In this case, lower concentrations (0.03–0.08%) of a 10% water extract of Ulva had no effect on seed germination and stimulated root growth, while higher concentrations (≥0.3%) had no effect on seed germination but inhibited root growth. The study further used Arabidopsis loss-of-function mutants in ABA hormone signalling and perception and demonstrated that the root growth promoting effect of lower concentrations of Ulva algal extract cannot be attributed to the changes in endogenous ABA signalling. However, ABA signalling contributes partially to the inhibitory effects associated with higher concentrations of a particular Ulva algal extract on primary root growth. Using auxin-, ethylene- and cytokinin-insensitive mutants, the authors also showed that ethylene, auxin and cytokinin hormone signalling pathways do not participate in the root growth stimulation associated with the application of Ulva algal extract. It was suggested that different mechanisms mediate germination induced by Ulva extract in Arabidopsis and that hormones such as ABA may contribute to germination inhibition provoked by these extracts in Arabidopsis [143].

The functional studies with loss-of-function mutants above highlight that trace amounts of hormones that are found in some (but not all) seaweed extracts are not responsible for the plant growth effects observed. It is possible that other unique chemical components abundantly present in seaweeds may induce endogenous growth hormone signalling as a part of other regulatory networks to modulate plant growth. Which specific compounds in seaweed extracts are accountable for eliciting this effect requires in-depth investigation.

### 3.2. Seaweed Derived Polysaccharides Induce Molecular Level Changes That Influence Plant Growth

Numerous studies are now reporting that “pure” seaweed extracts rich in polysaccharides can act as metabolic enhancers to influence growth in plants [144,145]. The investigation of the biological effects induced by seaweed carbohydrates on plant systems is a newly emerging area of research [11]. SWEs are a rich source of carbohydrates with high biodiversity, largely classified based on different classes of brown, green and red seaweeds. Brown seaweeds (phylum Ochrophyta) commonly contain laminarin and mannitol as storage carbohydrates, and fucoidans, cellulose, sulphated xylofucoglucan, sulphated xylofucoglucuronan, β-(1-3)-glucans and alginates as cell wall carbohydrates [32]. Green seaweeds (phylum Chrorophyta) mostly use starch, inulin (fructan) and sucrose as storage carbohydrates, and ulvans, cellulose, xyloglucan, mannans, glucuronan and β-(1-3)-glucan as cell wall carbohydrates. Red seaweeds (phylum Rhodophyta) storage carbohydrates include floridean glycogen, starch, mannitol, floridoside and isofloridoside. The cell walls in red seaweed comprise agars, carrageenans, cellulose, glucomannan, other mannans and xylans [32,146]. The modes of action of seaweed extracts are not clearly understood. However, plant priming and seaweed extract elicited responses have several common attributes [144], and seaweed-derived carbohydrates namely laminarin, fucoidan, alginate, carrageenan, ulvan or other non-carbohydrate compounds can potentially serve as molecular priming agents or key bioactives in SWEs to elicit discrete plant responses in treated plants [11]. However, the difference in the type of seaweed raw material, extraction parameters, time, temperature, etc., can influence the composition of extracts and the structural and functional integrity of polysaccharides contained therein, which in turn can give rise to different effects on treated plants [39].

Depending on the extraction methodology used and the quantity and availability of bioactives present, seaweed extracts can exert differential effects with respect to plant growth and stress tolerance [39,40,41]. Alginates are one of the major components of brown seaweed cell walls. Interestingly, depolymerised alginates (0.5–1 mg mL^−1^) have been shown to enhance growth in rice and peanut plants cultivated hydroponically [147]. Additionally, a mixture of oligo-alginates stimulated growth of lettuce roots [148] and elongation of carrot and rice roots [149]. Another group of major cell wall polysaccharides found in red seaweeds are carrageenans. Several studies have shown that carrageenans and their oligo forms can induce growth in plants ([150] and references therein). Extracts prepared from seaweed are also rich in polysaccharides such as laminarin, fucoidan (brown seaweed) and ulvans (green seaweed). Detailed investigations are still required to delineate the individual and synergistic roles, if any, played by seaweed derived polysaccharides and other components in improving cell division and plant growth.

## 4. Transcriptome and Metabolome Changes in Plants Treated with Seaweed Extracts

Several gene level investigations and traditional microarray platforms with a limited number of genes in the array have identified transcript level changes occurring in plants in response to seaweed extract applications [151]. A lower concentration of *A. nodosum* derived extract increased plant biomass and modulated the phenylpropanoid and flavonoid pathways to stimulate the growth as well as the nutritional quality of spinach in an in vitro experiment performed on half-strength Murashige and Skoog basal medium [152]. An acid extract from freshly harvested *A. nodosum* boosted total dry weight in rapeseed (*Brassica napus*) grown in the nutrient solution [153]. This significant increase in shoot and root weight was correlated with an enhanced nitrate uptake caused by the SWE treatment [153]. The differentially expressed genes associated with the SWE treatment in this study were related to the modulation of endogenous hormones, photosynthesis and nutrient uptake and transport [151,153].

On the other hand, recent studies using omics technologies such as transcriptomics and metabolomics have provided a holistic understanding of the diverse range of complex physiological and cellular processes that take place in plants as a result of seaweed extract treatment, and fundamental insights into the regulatory pathways involved in such events (reviewed by [132,154]). An in-depth transcriptome, metabolome and lipidome analysis performed on samples collected from *A. nodosum* extract SuperFifty (SF) pretreated Arabidopsis plants showed significant upregulation of specific photosynthesis, hormone signalling and growth-related genes both in control and oxidative stress conditions, providing information on how growth is stimulated by this specific seaweed extract [155]. A functional and healthy shoot apical meristem (SAM) is vital for plant vegetative growth and for transition to reproductive growth [156]. RNA in situ hybridisation analysis of SAM derived from SF treated Arabidopsis plants under non-stress and drought stress conditions showed high expression of cell cycle marker gene *HISTONE H4* (*HIS4*) in the newly dividing SAM tissues in both groups, which provides evidence that active cell division occurs in the SAM of plants treated with the SF [144].

## 5. Seaweed Extract Induced Stress Mitigation

Stress tolerance is perhaps the most important benefit of biostimulants in plants. Not surprisingly, various positive effects of biostimulant formulations against a broad range of abiotic stress conditions such as drought, temperature deviations (both heat and cold), salinity, accumulation of reactive oxygen species (ROS), mechanical injuries and chemical toxicity are among the most widely reported features of these products [17]. Efforts to elucidate the mechanisms through which seaweed extracts induce stress mitigation span several decades and include both classic and modern approaches. For example, as early as the 1980s, it was hypothesised that a major factor contributing to the beneficial effects of seaweed treatments is their cytokinin content. However, experiments with the seaweed concentrate “Kelpak” and potassium stressed wheat suggested that these phytohormones are not the only responsible organic bioactive compounds [157]. Zhang and Ervin [158] speculated that SWE containing 66 µg g^−1^ zaetine ribozide (ZR) is able to induce the endogenous cytokinin levels of creeping bentgrass (*Agrostis stolonifera*), as well as foliar tocopherol and root mass, which in turn may lead to increased drought tolerance. The same authors also investigated the impact of two SWEs and an exogenous ZR standard against long-term heat stress in *A. stolonifera* [159]. They found that all three applications similarly enhanced endogenous ZR levels and superoxide dismutase activity and reduced the decline in photochemical efficiency and root viability. Thus, it appears that modulation of endogenous phytohormones in seaweed biostimulant treated plants is important both for plant performance under normal conditions (as discussed above) and under stress.

### 5.1. Seaweed Extracts Induce Plant Priming

**Figure 2:**
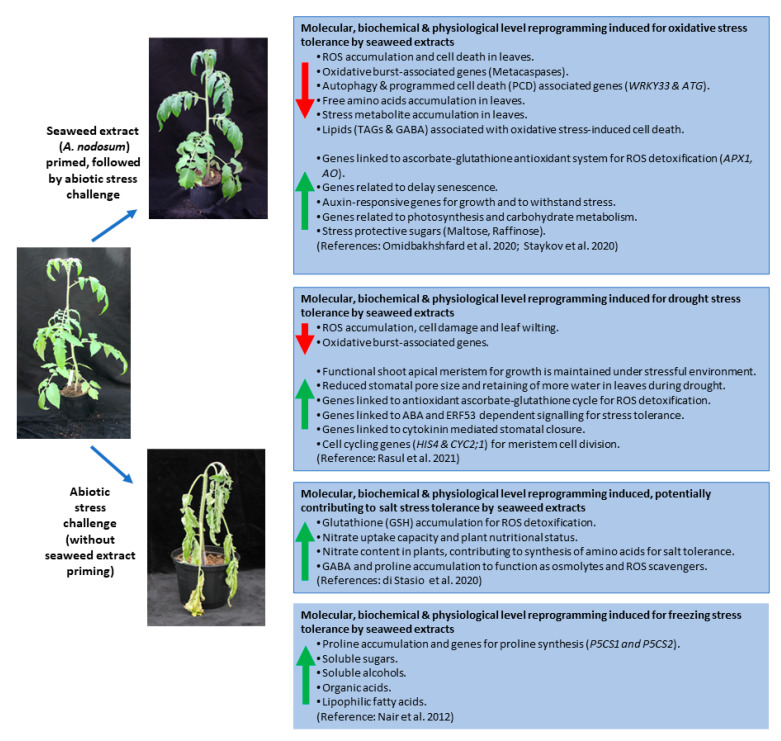
Commercially available *A. nodosum*-based seaweed extracts are proven to induce plant priming and abiotic stress tolerance in the model plant Arabidopsis and in crops. Molecular, biochemical and physiological level stress adaptations that occur in seaweed based biostimulant primed plants during oxidative, drought, salt and freezing stress challenges are also summarised above [144,155,165,166,167]. The tomato plants in the figure were primed with *A. nodosum* extract, SuperFiffty, SF or untreated, with both treatment groups subsequently exposed to drought stress. Red and green arrows show processes, genes, metabolites that are repressed/downregulated and activated/upregulated, respectively.

Certain natural or synthetic compounds, when exogenously applied, have the potential to change the physiological status of plants to a “primed state”. These pretreated or primed plants will respond more rapidly and/or more robustly upon subsequent biotic and abiotic stress exposures [11,160,161,162]. The priming process is mediated at the cellular level by epigenetic and chromatin-based mechanisms [163] and is mostly maintained or memorised by the plant for a few days to several weeks [164]. A growing body of evidence suggests that plant priming and seaweed extract elicited responses have several common attributes. Newer omics level analyses and integrated systems biology approaches have been employed in recent studies to elucidate the complex molecular mechanisms underlying the stress tolerance phenotypes observed in plants following seaweed extract treatment. This has now provided a highly informative snapshot of the range of complex physiological and cellular processes and functions occurring in biostimulant treated plants exposed to stress conditions (Figure 2).

### 5.2. Seaweed Extracts-Based Abiotic/Oxidative Stress Reduction

In a comprehensive study by Omidbakhshfard et al. [155], transcriptomic, metabolomic, and lipidomic data were collectively used to decipher the molecular mechanisms of oxidative stress tolerance induced by the seaweed biostimulant SF in Arabidopsis. In this experiment, SF was used in a foliar pretreatment step to prime the plants. Subsequently, the oxidative stress inducing agent paraquat (PQ) was applied to generate ROS levels *in planta*, similar to those which occur during abiotic stress events. SF based plant priming fully protected Arabidopsis from oxidative stress, as evident from the complete lack of damage on the leaves in SF-pretreated plants sprayed with PQ. At the molecular level, this was confirmed by measuring ROS marker genes, which were highly induced by the oxidative stress but not in the SF-pretreated plants exposed to subsequent PQ application. Furthermore, genes involved in autophagy and in ROS-induced programmed cell death were activated by the oxidative stress alone but not in the SF-pretreated plants exposed to oxidative stress ([155]; Figure 2). The detailed study of transcriptomes by RNA-seq identified genes related to photosynthesis, growth, and auxin signalling induced in primed PQ-treated plants, as compared to those subjected to oxidative stress alone. The better growth and photosynthesis of SF pretreated plants may explain the mitigation of oxidative stress at the molecular level. Analysis of primary metabolites by GC-MS identified accumulation of high levels of the stress metabolite gamma-aminobutyric acid (GABA) and several amino acids in the stressed plants. GABA is a typical stress metabolite, which also retards growth, while the accumulation of amino acids is characteristic of protein degradation. These observations are consistent with the transcriptome analysis, indicating growth inhibition and activation of autophagy. These molecular signatures are completely absent in SF pretreated plants. Furthermore, they accumulate some organic acids and stress protective metabolites such as maltose and raffinose, which may collectively contribute to the stress mitigating effect of SF at the molecular level ([155]; Figure 2). The lipidomic assessment conducted by this team indicated that lipids associated with oxidative stress-induced cell death and chloroplast degradation, such as triacylglycerols (TAGs), declined in the SF pretreated plants. The beneficial effect of SF priming on reducing oxidative stress symptoms was further confirmed in the two important crops tomato and pepper, which were compared to Arabidopsis ([165]; Figure 2). In this study, quantification of primary metabolites showed accumulation of stress metabolites in plants exposed to oxidative stress alone but not in plants treated with SF and subsequently subjected to oxidative stress. Furthermore, the metabolome analyses identified characteristic metabolite signatures induced by SF in the three investigated species ([165]; Figure 2).

### 5.3. Seaweed Extract-Based Plant Priming and Drought Stress Mitigation

In another study focused on the protective effect of SF against drought stress in Arabidopsis, Rasul et al. [144] demonstrated how SF counteracts long-term dehydration at the molecular level. Drought stress alone resulted in growth cessation, wilting, damage on the leaves, accumulation of hydrogen peroxide and induction of ROS marker genes. Furthermore, it induced the expression of the stress-responsive negative growth regulator *RESPONSIVE TO DESICCATION 26* (*RD26*) and completely switched off the expression of the cell cycle marker gene *HISTONE H4* (*HIS4*), crucial for maintaining the shoot apical meristem. However, in SF-treated plants, none of these negative effects were observed, plants continued to grow, and the expression of *HIS4* was maintained even after 11 days of drought ([144]; Figure 2). In this study, it was observed that SF priming triggered a timely reduction in stomatal aperture, helping to maintain tissue water levels under drought. The ABA-dependent signalling components *RCAR3* and *RBOHD* as well as the cytokinin-mediated signalling components *cytokinin response regulator 2* (*ARR2*) and *apoplastic peroxidase* (*PRX34*), which are important for stomatal closure during stress challenges, were modulated in SF-primed plants ([144]; Figure 2). In a similar fashion, priming Arabidopsis plants with a commercially available acid extract of *A. nodosum* (ALGEA) downregulated the expression of the transcription factor *AtMYB60*, which is potentially involved in the regulation of stomatal movement [168].

### 5.4. Seaweed Extract-Based Plant Priming for Salt and Freezing Induced Damage

SF-based plant priming has been demonstrated to remodel leaf nitrogen metabolism in response to salinity and/or osmotic stress in tomatoes. Improved nutrient uptake and limited cellular damage caused by ROS accumulation, as well as increased glutathione in SF treated plants has been proposed to prevent and/or repair damages caused by oxidative stress under salinity conditions ([41,166]; Figure 2). In turn, a lipophilic fraction of *A. nodosum* (Acadian) was shown to enhance freezing tolerance in Arabidopsis. Soluble sugars, sugar alcohols, organic acids and lipophilic components such as fatty acids accumulated in seaweed extract treated plants and enhanced the expression of genes coding for proline synthesis (*P5CS1* and *P5CS2*) ([167]; Figure 2).

Overall, the specific studies mentioned above identify genes and metabolites that collectively contribute to abiotic tolerance induced by the seaweed biostimulants. Additionally, studies suggest that compounds such as oligosaccharides and polysaccharides may potentially be responsible for the priming and biostimulatory effects, with other components of the SWEs potentially contributing synergistically. Further functional studies are needed to confirm the roles of specific genes that are modulated, as well as to identify which exact components from seaweed biostimulants exert the specific protective effects.

## 6. Improvements in Nutrient Use Efficiency, Productivity and Quality Traits Induced by Seaweed Extracts

Improving resource use efficiency (RUE), while reducing the overuse of chemical fertilisers such as nitrates, is an important target to be achieved for enhancing the sustainability in agricultural systems [6,169]. *A. nodosum* extracts have been shown to ameliorate nutrient uptake in plants. Treatment with an *A. nodosum* extract SF in tomatoes (*Solanum lycopersicum* cv. MicroTom) improves nitrogen use assimilation by increasing the antioxidant glutathione (GSH), nitrate and alanine contents in tomato plants [41]. SF treatment also completely reshaped the free amino acids profile of tomato leaves under non-stressful conditions and seaweed treated plants had leaves enriched with total, free and minor amino acids [41]. Minor amino acids can function as compatible metabolites and antioxidants in leaves and can be exported to fruits, contributing to increases in the nutritional value and antioxidant properties of tomato fruit [170] and potentially improving shelf life [171]. These pre-adaptation responses may lead to enhanced tomato growth and yield observed following timely applications of SF both under non-stressful and stressful field conditions [167,170]. Ameliorated nitrogen uptake and assimilation after using an *A. nodosum* extract was also demonstrated in barley [172]. The *A. nodosum* treatment in spinach upregulated genes coding for glutamine synthetase (*GS1*), which catalyses the conversion of inorganic nitrogen (ammonium) to organic form (glutamine) and plays an essential role in nitrogen metabolism and assimilation [152]. The treatment also promoted the protein synthesis in spinach and increased the activity of nitrate reductase, an enzyme that catalyses the reduction of nitrate to nitrite, which is the first step of the nitrate assimilation pathway [152,173]. An increased tissue concentration of Mg, Mn, Na, and Cu and root to shoot translocation of the micronutrients Fe and Zn was confirmed in oilseed rape plants grown in a nutrient solution supplied with a 0.1% *v*/*v A. nodosum* extract [174]. Microarray analysis and gene expression data showed an increased expression in seaweed extract treated plants of the Cu transporter *COPT2* and the *NRAMP3* which has a putative a role in Fe and Zn translocation [174]. The *A. nodosum* extract treatment upregulated genes involved in the transport of amino acids (*LHT1* and *AAP5*), calcium (*CAX3*, *CAX7* and *ACA1*), peptides (*ATOPT3*), nucleotide sugar derivatives (*UTR2* and *UTR3*), copper (*COPT2*), nitrate (*NRT1.5*), nucleotides (*ATPUP10*), sugars (*MSS1*), and sulphate (*SULTR1*, *SULTR3*, and *AST56*) in Arabidopsis [172]. The above-mentioned studies concluded that the mineral content from the seaweed extracts is negligible and insufficient for inducing these effects. Instead, the seaweed extract treatments may act on plant metabolism and signalling pathways specifically associated with nutrient uptake and/or translocation. However, further studies are required to determine how biostimulants and their components interact with intra- and extra-cellular signalling components in plants that are associated with the induction of the observed interlinked beneficial effects regarding nutrient uptake and plant growth.

## 7. Conclusions

The accurate application of seaweed-based biostimulant technologies in modern agriculture, with minimal environmental impacts, depends on a number of key factors. Firstly, the use of renewable seaweed resources as raw materials for biostimulants must be undertaken in a manner that is sustainable and includes defined harvesting plans, rotational periods, monitoring to assess regeneration post-harvesting and measures to ensure that potential effects on marine and coastal habitats and species are prevented or minimised. Secondly, the use of refined manufacturing practices that have been developed on the basis of an in-depth understanding of the molecular mechanisms through which these extracts induce desired benefits in treated crops, is essential in order to deliver consistent and reliable products to end users. Recent studies have shown that pure seaweed extract-based biostimulants can prime model plants and crops. The main concept underlying molecular priming is that timely application of specific seaweed extracts modulates genes and metabolic pathways in plants and provides durable tolerance against abiotic and oxidative stresses. Thus, based on the weather forecast, forthcoming unfavourable events can be predicted, which allows growers to respond by applying SWE biostimulants beforehand to induce the cellular defence mechanisms and secure stress protection. Additionally, seaweed extract-based priming methodologies have been shown to bring about desired changes to plant signalling cascades and metabolic processes, specifically to induce beneficial effects in terms of improved plant growth and crop performance. The precise mode(s)-of-action activated by seaweed extracts and their individual bioactive compounds, and/or the synergies potentially responsible for these effects in plants, require further functional studies at the molecular level.

## Figures and Tables

**Table 1:** Estimated wild seaweed and aquatic plants production worldwide in 2019, based on FAO global capture production statistics [42].

No.	Country	Category	Tonnes (Live Weight)					
			Brown Seaweed	Red Seaweed	Green Seaweed	Seaweed Nei	Aquatic Plants Nei	Total
1	Chile	† Bull kelp (*Durvillaea antarctica*), *Lessonia trabeculata*, Chilean kelp (*Lessonia nigrescens*), Giant kelps nei, (e.g., *Macrocystis pyrifera*). ‡ *Chondracanthus chamissoi*, *Gymnogongrus furcellatus*, *Mazzaella laminarioides*, *Gelidium* spp. *(Gelidiaceae)*, *Gracilaria* spp. (Gracilariaceae), Leister (*Sarcothalia crispate*), Nori nei (*Porphyra* spp.), Skottsberg’s Gigartina *(Gigartina skottsbergii)*. # Aquatic plants nei.	288,486.00	115,973.00			467.00	404,926.00
2	China	# Aquatic plants nei					174,450.00	174,450.00
3	Norway	† Babberlocks *(Alaria esculenta)*, Brown seaweeds (general), North Atlantic rockweed *(Ascophyllum nodosum)*, North European kelp (Laminaria hyperborea). * Gut weed *(Ulva intestinalis).*	162,824.00		128.00			162,952.00
4	Japan	† Japanese kelp *(Laminaria japonica).* # Aquatic plants nei.	46,500.00				20,300.00	66,800.00
5	France	† North European kelp *(Laminaria hyperborea)*, Tangle *(Laminaria digitata)*, Sea thong *(Himanthalia elongata)*. ‡ Dulse *(Palmaria palmata)*, Giant gelidium *(Gelidium corneum)*, Red seaweeds (general). # Other Seaweeds nei.	51,141.92	158.12		0.01		51,300.05
6	Indonesia	‡ Red seaweeds (general).		44,500.00				44,500.00
7	Peru	† *Lessonia trabeculata*, Chilean kelp *(Lessonia nigrescens)*, Giant kelp *(Macrocystis pyrifera)*. ‡ *Chondracanthus chamissoi*.	34,836.78	1511.00				36,347.78
8	Ireland	† North Atlantic rockweed *(Ascophyllum nodosum)*, North European kelp *(Laminaria hyperborea)*. ‡ Red seaweeds (general).	29,400.00	100.00				29,500.00
9	India	† Brown seaweeds (general) * Green seaweeds (general). ‡ Red seaweeds (general).	3219.28	4136.78	11,043.93			18,399.99
10	Iceland	† North Atlantic rockweed *(Ascophyllum nodosum)*, North European kelp *(Laminaria hyperborea)*, Tangle *(Laminaria digitata)*.	17,533.00					17,533.00
11	Morocco	‡ Red seaweeds (general).		17,317.71				17,317.71
12	Canada	† North Atlantic rockweed *(Ascophyllum nodosum)*.	12,655.00					12,655.00
13	South Africa	† Brown seaweeds (general). ‡ *Gelidium* spp. (Gelidiaceae).	8265.00	735.00				9000.00
14	Russia	† North European kelp *(Laminaria hyperborea)*, Brown seaweeds (general). ‡ Red seaweeds (general). # Aquatic plants nei.	8968.00	1.00			2.00	8971.00
15	Rep. of Korea	† Japanese kelp, Wakame *(Undaria pinnatifida)*, Brown seaweeds (general). ‡ *Gracilaria* spp. (Gracilariaceae), Laver (Nori, *Porphyra tenera)*. * Fragile codium *(Codium fragile)*, Green laver *(Monostroma nitidum)*. # Other Aquatic plants nei.	4290.00	75.00	1060.00		3285.00	8710.00
16	Mexico	† Brown seaweeds (general). ‡ Red seaweeds (general).	5291.23	2034.41				7325.64
17	Spain	† Wakame *(Undaria pinnatifida)*, Brown seaweeds (general). ‡ *Gelidium* spp. (Gelidiaceae), Red seaweeds (general), Ribboned nori *(Porphyra linearis)*. * Green seaweeds (general). # Seaweeds nei.	314.90	242.29	0.01	2595.09		3152.29
18	USA	† Giant kelps nei, (e.g., *Macrocystis pyrifera)*. * Green seaweeds (general).	6.00		3125.00			3131.00
19	Australia	† Brown seaweeds (general).	1923.00					1923.00
20	Italy	‡ Red seaweeds (general). * Green seaweeds (general).		400.00	800.00			1200.00
21	Portugal	‡ Red seaweeds (general).		1111.49				1111.49
22	Madagascar	‡ Red seaweeds (general).		800.00				800.00
23	New Zealand	‡ *Pterocladia lucida*. * Sea lettuces nei. # Seaweeds nei.		0.64	0.01	508.81		509.46
24	Philippines	‡ Red seaweeds (general)		364.53				364.53
25	Taiwan	‡ *Gelidium* spp. (Gelidiaceae), Laver (Nori; *Porphyra tenera)*. * Lacy sea lettuce *(Ulva pertusa)*. # Aquatic plants nei.		116.71	73.13		112.64	302.48
26	Estonia	‡ Red seaweeds (general).		60.00				60.00
		Total	675,654.11	189,637.68	16,230.08	3,103.91	198,616.64	1,083,242.42

† Brown seaweeds, ‡ red seaweeds, * green seaweeds, # undefined aquatic plants or seaweeds. Scientific names and FAO official common names in English are provided, where available.

## Data Availability

Not applicable.
